# Uso de diálisis de único paso con albúmina en paciente con falla hepática aguda por dengue: reporte de caso

**DOI:** 10.7705/biomedica.7682

**Published:** 2026-06-01

**Authors:** Carlos Carvajal, Juan Arias, Santiago Torres

**Affiliations:** 1 Departamento de Cuidado Crítico de Adulto, Hospital Pablo Tobón Uribe, Medellín, Colombia Hospital Pablo Tobón Uribe Medellín Colombia; 2 Departamento de Anestesiología y Reanimación, Universidad de Antioquia, Medellín, Colombia Universidad de Antioquia Universidad de Antioquia Medellín Colombia

**Keywords:** falla hepática, dengue, dengue grave, terapia de reemplazo renal continuo, diálisis, albúminas, Liver failure, dengue, severe dengue, continuous renal replacement therapy, dialysis, albumins

## Abstract

El dengue es la infección arboviral más frecuente a nivel mundial y hasta el 5 % de los pacientes pueden desarrollar formas graves con compromiso multiorgánico y mayor riesgo de muerte.

Se presenta el caso de una paciente de 39 años, procedente de zona endémica y residente en área urbana, que consultó por fiebre de cinco días asociada a mialgias y sangrado vaginal. Al ingreso se documentó trombocitopenia grave, disfunción hepática y disfunción renal. A pesar del tratamiento inicial con cristaloides, tuvo deterioro clínico progresivo de su enfermedad, lo que resultó en falla hepática aguda y progresión de la lesión renal aguda, con necesidad de iniciar soporte ventilatorio invasivo, vasopresores y terapia con diálisis de albúmina de único paso más hemodiafiltración veno-venosa continua. Hubo mejoría clínica con recuperación progresiva de la función hepática y renal, y fue trasladada a hospitalización después de doce días en cuidados intensivos.

Este caso destaca la posible utilidad de esta terapia en pacientes con falla hepática aguda en sitios sin acceso a técnicas avanzadas de soporte hepático o cuando existe alguna contraindicación para el trasplante hepático.

## Abstract (en)

Dengue is the most common arboviral infection worldwide, and up to 5% of patients may develop severe forms with multiorgan involvement and an increased risk of death.

We present the case of a 39-year-old woman from an endemic area, living in an urban setting, who presented with five days of fever associated with myalgias and vaginal bleeding. On admission, severe thrombocytopenia and acute hepatic and renal dysfunction were documented. Despite initial treatment with crystalloids, her condition progressively worsened, leading to acute liver failure and worsening acute kidney injury, requiring invasive mechanical ventilation, vasopressor support, and single-pass albumin dialysis plus continuous veno-venous hemodiafiltration. The patient showed clinical improvement with progressive recovery of liver and kidney function and was transferred to general hospitalization after 12 days in intensive care.

This case highlights the potential usefulness of this therapy in patients with acute liver failure in settings without access to advanced liver support systems or when liver transplantation is contraindicated.

El dengue es la infección arboviral más común a nivel mundial, con una estimado de alrededor de 50 a 400 millones de infecciones anuales, aproximadamente, en 100 países tropicales y subtropicales ^1^^,^^2^. En las Américas, la transmisión representa un importante problema de salud pública, favorecido por la elevada infestación del mosquito *Aedes aegypti*, principal vector transmisor, de tal forma que cerca de 500 millones de personas están en riesgo de sufrir la enfermedad ^2^^,^^3^.

En la región, Colombia es uno de los países más afectados, observándose casos durante todo el año, con una incidencia para el 2021 de 172,9 casos por cada 100 000 habitantes en riesgo, con algunas regiones con incidencias superiores a 400 casos por cada 100 000 habitantes en riesgo. Cerca del 51,6 % de los casos reportados de dengue a nivel nacional presentaron signos de alarma y el 2,1 % se clasificaron como dengue grave, los cuales requirieron hospitalización ^2^^,^^4^.

El virus del dengue pertenece a la familia Flaviviridae y se conocen cuatro serotipos, los cuales circulan alternativamente en Colombia ^2^. Desde el punto de vista fisiopatológico, al parecer, existe una lesión celular directa a nivel endotelial con disfunción del glicocálix que orquesta efectos sobre la permeabilidad microvascular y los mecanismos de regulación de la trombosis ^5^.

El espectro clínico del dengue es amplio, abarcando desde infecciones asintomáticas hasta formas graves con compromiso multiorgánico y mayor riesgo de morir ^6^^,^^7^. Aunque no existe un tratamiento específico para el dengue, hay suficiente evidencia clínica que respalda que un manejo hidroelectrolítico adecuado y oportuno reduce el riesgo de complicaciones y mortalidad ^5^. En los casos más graves puede ser necesario el soporte orgánico, incluyendo terapias extracorpóreas cuando hay disfunción hepática grave ^8^.

A continuación, se presenta el caso de una paciente previamente sana, con infección por virus del dengue, que desarrolló falla multisistémica con falla hepática hiperaguda que requirió tratamiento con diálisis de único paso con albúmina, con lo cual se logró una buena respuesta clínica.

## Descripción del caso

Se trata de una mujer de 39 años, residente del área urbana de una zona endémica de Colombia, dedicada a actividades de vigilancia, sin antecedentes médicos relevantes, que fue remitida desde un hospital de segundo nivel en Medellín, por cuadro de cinco días de evolución de fiebre de inicio súbito, cefalea frontal, mialgias, dolor abdominal, náuseas sin vómito y sangrado gingival y vaginal.

Inicialmente, presentó episodios de lipotimia con alteración progresiva del estado de conciencia, lo que motivó la consulta. Al ingresar a urgencias, la paciente se encontraba somnolienta, tenía fiebre con una temperatura de 39,2 °C, presentaba palidez, cifras de presión arterial bajas, sin linfadenopatías asociadas, erupciones petequiales ni signos de enfermedad hepática crónica. Su frecuencia cardíaca era de 118 latidos por minuto y su presión arterial de 85/35 mm Hg. El abdomen estaba blando, no presentaba dolor y no tenía hepatomegalia ni esplenomegalia. No había signos de irritación meníngea.

Los estudios iniciales de laboratorio tomados en el sitio de remisión informaban prolongación de los tiempos de coagulación, elevación de las transaminasas, hiperbilirrubinemia a expensas de la bilirrubina directa, disfunción renal y trombocitopenia grave asociada a episodios de sangrado, predominantemente gingival y vaginal. Ante el cuadro clínico, se decidió su hospitalización.

En urgencias se sospechó dengue con signos de alarma, lo que posteriormente se confirmó con el antígeno NS1 positivo y pruebas serológicas. Se inició la administración de cristaloides y se hicieron ajustes según los signos vitales, la diuresis y el hematocrito, además de transfusiones por trombocitopenia grave y sangrado vaginal persistente.

A las 24 horas de estar hospitalizada presentó un mayor deterioro clínico con necesidad de traslado a la unidad de cuidados intensivos para respiración mecánica asistida e inicio de soporte vasoactivo. Se decidió practicarle monitoreo invasivo con línea arterial y catéter venoso central y, dada la mayor disfunción hepática, fue remitida al Hospital Pablo Tobón Uribe.

En la ecografía abdominal se observó hepatomegalia con esteatosis difusa y derrame pleural bilateral, sin alteraciones de la vía biliar ni a nivel de útero ni de otros órganos anexos. La tomografía de cráneo no evidenció signos de hipertensión endocraneana.

Al ingreso a cuidados intensivos estaba profundamente sedada, con respiración mecánica asistida por hipoxemia moderada y con requerimiento de norepinefrina.

Se documentó falla hepática aguda y disfunción renal con falla renal aguda KDIGO-3 (*Kidney Disease Improving Global Outcomes*). El APACHE II (*Acute Physiology and Chronic Health Disease Classification System* II) al ingreso fue de 17 puntos. Se decidió iniciar terapia de reemplazo renal continua con hemodiafiltración veno-venosa constante con altos volúmenes, acetil cisteína en infusión (150 mg/kg en 60 minutos, luego 12,5 mg/kg/hora en 4 horas y finalmente 6,125 mg/kg/hora por 72 horas) y, en conjunto con los servicios de nefrología y hepatología, se comenzó diálisis con albúmina de un solo paso, con una máquina de diálisis Prismaflex, Baxter™, con un filtro Prismaflex ST150.

**Figura 1.**
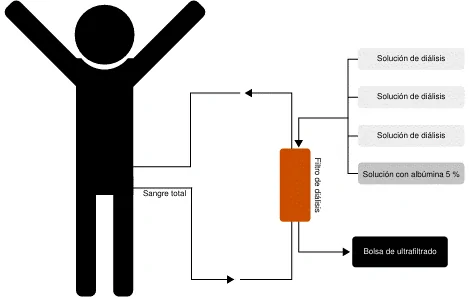
Esquema de diálisis con albúmina de paso único

**Figura 2.**
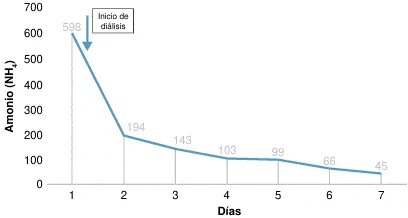
Evolución de los valores de amonio (NH_4_) en una paciente con dengue grave con compromiso renal manejada con diálisis con albúmina de paso único, agosto de 2024

La prescripción de la terapia de reemplazo renal se realizó con el objetivo de alcanzar una dosis de diálisis de 35 ml/kg/hora (bomba de sangre: 180 ml/minuto; dializante: 1000 ml/minuto; antes de la dilución: 1000 ml/minuto y después de la dilución: 450 ml/minuto). Se administró por 5 horas una infusión de 1000 ml/hora de dializante cada 24 horas, y un recambio con albúmina al 5 % (3750 ml de solución 4K más 1250 ml de albúmina al 20 %), terapia que se mantuvo por cuatro días consecutivos y que se ilustra en la figura 1, con lo cual se evidenció una depuración del amonio (NH_4_), como se muestra en la figura 2.

Se descartaron otras infecciones, como se muestra en el cuadro 1 y la familia manifestó que la paciente no tomó medicamentos hepatotóxicos ni remedios tradicionales.

**Cuadro 1.** Evolución de la bioquímica hepática, plaquetas y tiempo de protrombina (*international normalized ratio*, INR) en los primeros 9 días de evolución, agosto del 2024

Fecha	Día 1	Día 2	Día 3	Día 4	Día 5	Día 6	Día 7	Día 8	Día 9
AST (U/l)	25 260	17 890	9219	3129	1598	979	744	524	364
ALT (U/l)	5191	3022	2307	1727	1298	1022	914	731	539
Bilirrubina total (mg/dl)	7,4	10,6	14,4	15,4	14,1	18,56	18,48	10,1	5,26
Bilirrubina directa (mg/dl)	3,85	6,93	11,98	13,34	10,76	11,71	11,13	8,33	6,23
F fosfatasa alcalina (U/l)	229	267	200	177	205	231	--	203	229
GGT (U/l)	--	384	--	325	--	--	--	--	--
Tiempo de protrombina (s)	20,1	19,1	19,1	17,1	15,5	14,7	14,7	14,8	13,1
*International normalized ratio*	2,1	2,0	1,72	1,59	1,4	1,32	1,32	1,33	1,18
Plaquetas por μl	20 000	89 000	89 000	120 000	100 000	66 000	63 000	65 000	61 000
Lactato (mmol/l)	4,7	3,2	4,3	2,3	1,9	2,3	2,1	2,2	1,5
PaO_2_/FiO_2_	77	93	82	140	246	278	410	403	414

ALT: alanino aminotransferasa; AST: aspartato aminotransferasa; GGT: gamma-glutamiltransferasa; PaO_2_/FiO_2_: índice PAFI o índice de Kirby

Se le practicó aspirado traqueal al quinto día por sospecha de neumonía asociada a la respiración mecánica asistida, aislándose *Pseudomonas aeruginosa* multisensible, con lo cual la paciente completó tratamiento antibiótico dirigido por siete días. Posteriormente, presentó mejoría clínica progresiva: estabilización hemodinámica, recuperación de la función hepática y renal, y mejoría de los índices de oxigenación, tal y como se muestra en el cuadro 2.

**Cuadro 2.** Perfil para búsqueda de las coinfecciones virales durante los primeros tres días de permanencia en la unidad de cuidados intensivos

Prueba	Resultado		Unidad/interpretación
Anticuerpos contra la hepatitis C	0,09		Índice de absorbancia
Antígeno de superficie contra la hepatitis B	0,28		Índice de absorbancia
Anticuerpos centrales totales *core* contra la hepatitis B	0,06		Índice de absorbancia
Anticuerpos IgM *core* contra la hepatitis B	0,11		Índice de absorbancia
Anticuerpos IgM contra la hepatitis A	0,2		Índice de absorbancia
Antígeno NS1 contra el dengue	Positivo		Cualitativo
Anticuerpos IgG contra el dengue	Positivo		Cualitativo
Anticuerpos IgM contra el dengue	Positivo		Cualitativo
RT-qPCR para el dengue	Positivo		Cualitativo
Inmunoglobulina M contra *Leptospira* spp.	Negativo		Cualitativo
Inmunoglobulina G contra *Leptospira* spp.	Negativo		Cualitativo
Inmunoglobulina G contra citomegalovirus	Positivo		Cualitativo
Inmunoglobulina M contra citomegalovirus	Negativo		Cualitativo
Anticuerpos anti-HIV	No reactivo		Cualitativo
Anticuerpos por microaglutinación contra *Leptospira* spp.	Negativo		Cualitativo
PCR para *Leptospira* spp.	Negativo		Cualitativo
Anticuerpos IgG contra el virus de Epstein Barr	44,58		U/ml
Anticuerpos IgM contra la cápside del virus de Epstein Barr	0,09		índice de absorbancia

NS1: *non-structuralprotein 1;* RT-qPCR: *reverse transcription-quantitative* PCR; PCR: *polymerase chain reaction;* HIV: *human immunodeficiency virus*

Se desmontó la sedación y se extubó con buena tolerancia, permaneciendo con terapia de reemplazo renal intermitente por algunos días más. Doce días después se trasladó a sala general de hospitalización y, finalmente, se le dio de alta con recuperación parcial de la función renal. Los diagnósticos de egreso fueron: dengue grave con compromiso multiorgánico, falla hepática hiperaguda, lesión renal aguda KDIGO 3, trombocitopenia grave y neumonía tratada asociada por *P. aeruginosa*.

### Consideraciones éticas

El presente trabajo corresponde a un reporte de caso clasificado como investigación sin riesgo, según la Resolución 8430 de 1993. Se obtuvo consentimiento del Comité de Ética en Investigación del hospital. Se garantizó la confidencialidad de la información.

## Discusión

En este artículo se documenta un caso de dengue grave con compromiso multiorgánico, caracterizado por disfunción cardiovascular con choque hipovolémico por fuga capilar, encefalopatía, falla hepática hiperaguda, lesión renal aguda y afectación hematológica. La paciente respondió favorablemente al manejo intensivo, incluida la diálisis de albúmina de paso único como terapia puente a la restauración de la función hepática, recuperación parcial de la función renal y reversión de la disfunción de otros órganos.

El dengue es la arbovirosis más frecuente en el mundo, causado por un virus ARN de cadena sencilla del género *Flavivirus*, con cuatro serotipos descritos (DEN-1 a DEN-4). En las regiones endémicas como Colombia, donde circulan los cuatro serotipos, la infección secundaria por un serotipo heterólogo se ha asociado a mayor riesgo de formas graves por el fenómeno de la potenciación dependiente de anticuerpos ^1^. La serología positiva de las inmunoglobulinas G y M sugiere que este se trata de uno de esos casos.

Se estima que cada año ocurren entre 56 y 390 millones de infecciones por dengue a nivel mundial, con una mortalidad cercana a las 36 000 muertes anuales; la mayor carga se concentra en el sudeste asiático, el pacífico occidental y las Américas, donde el cambio climático y la urbanización favorecen la expansión del mosquito *Aedes*^6^^,^^8^^-^^10^.

En Colombia, cerca del 2 al 5 % de los casos puede evolucionar a dengue grave ^2^, siendo más frecuentes las complicaciones como choque hipovolémico por fuga capilar, sangrado masivo y falla orgánica múltiple. Factores como el sexo femenino y comorbilidades como la diabetes mellitus, la hipertensión arterial y la enfermedad renal crónica se asocian con mayor riesgo de desarrollo de formas graves ^5^, aunque en esta paciente previamente sana probablemente la respuesta inmunológica exacerbada fue determinante.

Según las guías del 2009 de la OMS (Organización Mundial de la Salud) ^7^, la infección por dengue se clasifica según el nivel de gravedad en: dengue sin signos de alarma, dengue con signos de alarma (dolor abdominal, vómitos persistentes, acumulación de líquidos, sangrado por mucosas, letargia, afectación hepática, aumento del hematocrito con disminución de las plaquetas), y dengue grave (dengue con fuga grave de plasma, sangrado grave o insuficiencia orgánica).

El curso clínico del dengue sigue típicamente una fase febril inicial, una fase crítica (cuando ocurre la fuga capilar) y una fase de recuperación ^5^.

En este caso, la paciente presentó signos iniciales de alarma y progresión rápida a dengue grave con choque hipovolémico, encefalopatía, coagulopatía grave, sangrado y compromiso renal.

La trombocitopenia y la coagulopatía en el dengue son multifactoriales: involucran supresión de megacariocitos, lisis plaquetaria mediada por anticuerpos y aumento del consumo por adherencia endotelial ^11^. La transfusión profiláctica de plaquetas sigue siendo controvertida, ya que la trombocitopenia aislada no siempre explica el sangrado que, en muchos casos, se relaciona más con la disfunción hepática y alteraciones de la coagulación ^11^^,^^12^.

Un hallazgo relevante fue la neumonía asociada a la respiración mecánica asistida por *P. aeruginosa*. Las infecciones intrahospitalarias en pacientes críticos con dengue son frecuentes. Se ha estimado que entre el 14 y el 44 % de las muertes por dengue pueden estar asociadas a coinfecciones bacterianas, especialmente bacteriemias y neumonías ^13^.

La afectación renal puede incluir alteraciones electrolíticas, hematuria y proteinuria. Sin embargo, se puede presentar compromiso grave que lleve a lesión renal aguda o a síndrome hemolítico urémico. Se ha descrito que la hipoperfusión renal y la necrosis tubular debidas a hipotensión, la lesión directa por el virus y la rabdomiólisis pueden estar involucradas en la fisiopatología de la disfunción renal ^14^^,^^15^.

El compromiso hepático guarda relación con la gravedad de la infección y es más frecuente en los casos de fiebre hemorrágica por dengue, especialmente en niños, en casos de infección previa y en presencia de trombocitopenia. Se reconoce que el mecanismo fisiopatológico combina la lesión hipóxica por choque, la lesión celular directa por el virus, la apoptosis y la respuesta inflamatoria mediada por los linfocitos CD4 y las citocinas ^16^^,^^17^. La mortalidad por falla hepática fulminante es alta y se agrava cuando no hay acceso oportuno al trasplante hepático.

Cuando hay disfunción hepática grave o falla hepática aguda -que puede poner al paciente en un riesgo de mortalidad de cerca del 50 %- es necesario ofrecer soporte vital multiorgánico, manteniendo una adecuada oxigenación y perfusión tisular, y considerar terapias de reemplazo hepático como puente a la recuperación ante la dificultad de acceso al trasplante.

Se han desarrollado varios sistemas basados en el concepto de diálisis de albúmina, y los más conocidos son: el sistema de recirculación de adsorbente molecular (MARS™, *Molecular Adsorbent Recirculating System*), el sistema de separación y adsorción de plasma fraccionado (FPSA, *Fractionated Plasma Separation and Adsorption*) Prometheus™ y el sistema de diálisis con albúmina de paso único. Esta última representa una alternativa viable, de menor complejidad técnica y costos más bajos frente a los otros dos sistemas mencionados, ya que adapta una terapia de reemplazo renal estándar para remover toxinas mediante un hemodlafiltro de alto flujo y una solución dializante con albúmina entre el 1 y el 5 %.

Aunque hay pocos reportes de diálisis con albúmina de paso único en pacientes con dengue grave, esta modalidad puede facilitar la depuración de toxinas como el amonio (NH_4_) y las bilirrubinas, ganando tiempo para la recuperación hepática espontánea ^8^^,^^18^^,^^19^.

Entre los mencionados reportes se encuentra el caso de una paciente de 10 meses en Tailandia, con dengue y falla hepática aguda, que recibió diálisis con albúmina de paso único combinada con intercambio plasmático y hemofiltración veno-venosa continua, con lo cual se logró darle de alta sin secuelas tras 72 días de hospitalización ^20^. También hay dos casos no publicados en ese país con uso de diálisis con albúmina de paso único en dengue grave ^21^.

En este caso, la implementación de la diálisis con albúmina de paso único junto con la hemodiafiltración continua y el soporte multiorgánico permitió la estabilización metabólica, contribuyendo a la mejoría clínica. Se optó por esta terapia ante la falta de terapias moleculares avanzadas, por su bajo costo y disponibilidad.

Finalmente, este caso destaca la importancia de reconocer tempranamente la progresión a dengue grave, optimizar la reanimación hidroelectrolítica y considerar terapias extracorpóreas como la diálisis con albúmina de paso único en los casos en los que se presente falla hepática aguda en entornos con recursos limitados.

Se requiere evidencia prospectiva y comparativa para establecer la eficacia y la seguridad de este tipo de diálisis como terapia puente en la falla hepática por dengue.

Como conclusión, en los casos de dengue grave con complicaciones renales, hepáticas, hematológicas u otras, que se presentan en entornos con limitados recursos, como en el presente caso, el manejo de soporte intensivo adecuado sumado al uso de diálisis con albúmina de paso único puede permitir una mejoría significativa de la función hepática y la estabilización hemodinámica de la paciente, lo cual sugiere que esta terapia puede ser una alternativa valiosa en estas situaciones. Sin embargo, se requiere del desarrollo de ensayos clínicos controlados que comparen y evidencien la costo-efectividad de las diferentes alternativas disponibles.
